# And Then There Was Light: Perspectives of Optogenetics for Deep Brain Stimulation and Neuromodulation

**DOI:** 10.3389/fnins.2017.00663

**Published:** 2017-12-12

**Authors:** Jean Delbeke, Luis Hoffman, Katrien Mols, Dries Braeken, Dimiter Prodanov

**Affiliations:** ^1^LCEN3, Department of Neurology, Institute of Neuroscience, Ghent University, Ghent, Belgium; ^2^Neuroscience Research Flanders, Leuven, Belgium; ^3^Life Science and Imaging, Imec, Leuven, Belgium; ^4^Environment, Health and Safety, Imec, Leuven, Belgium

**Keywords:** viral vectors, biosafety, optogoenetics, deep brain stimulation, neural prosthesis, Parkinson's disease, epilepsy, neuromodulation

## Abstract

Deep Brain Stimulation (DBS) has evolved into a well-accepted add-on treatment for patients with severe Parkinsons disease as well as for other chronic neurological conditions. The focal action of electrical stimulation can yield better responses and it exposes the patient to fewer side effects compared to pharmaceuticals distributed throughout the body toward the brain. On the other hand, the current practice of DBS is hampered by the relatively coarse level of neuromodulation achieved. Optogenetics, in contrast, offers the perspective of much more selective actions on the various physiological structures, provided that the stimulated cells are rendered sensitive to the action of light. Optogenetics has experienced tremendous progress since its first *in vivo* applications about 10 years ago. Recent advancements of viral vector technology for gene transfer substantially reduce vector-associated cytotoxicity and immune responses. This brings about the possibility to transfer this technology into the clinic as a possible alternative to DBS and neuromodulation. New paths could be opened toward a rich panel of clinical applications. Some technical issues still limit the long term use in humans but realistic perspectives quickly emerge. Despite a rapid accumulation of observations about patho-physiological mechanisms, it is still mostly serendipity and empiric adjustments that dictate clinical practice while more efficient logically designed interventions remain rather exceptional. Interestingly, it is also very much the neuro technology developed around optogenetics that offers the most promising tools to fill in the existing knowledge gaps about brain function in health and disease. The present review examines Parkinson's disease and refractory epilepsy as use cases for possible optogenetic stimulation therapies.

## 1. Introduction

Therapeutic use of electricity dates back to antiquity. Deep Brain Stimulation (DBS) originates from the advancement of the sterotactic surgical techniques, which allowed the transition from lesional to stimulating technique of the deep nuclei of the brain for therapeutic purposes. Readers are directed to the historical survey by Sironi (2011) for further information. At present, DBS is an established therapeutic option for a variety of neurological diseases, such as Parkinson's disease (Beitz, 2014), essential tremor (Børretzen et al., 2014), dystonia (Lumsden et al., 2013) and obsessive-compulsive disorder (Greenberg et al., 2010). Closely related to DBS is another electrically-based therapy called neuromodulation. Applied to peripheral (Bhadra and Peckham, 1997) or cranial nerves (Ben Menachem and French, 2005), the spinal cord (Francois et al., 2017), the cochlea (Rajguru et al., 2010), the retina (Nirenberg and Pandarinath, 2012) as well as to the brain (Rossi et al., 2016). This technique is used to treat many conditions and for theses indications it can already be classified as a well–accepted clinical treatment.

Using light to control neural activity holds promises for much improvement since genetics has developed the tools to make specific structures light sensitive. Under appropriate conditions, the action potential, an all-or-nothing phenomenon seen as the neural information carrier, can be triggered by light^1^ as well as by electrical stimulation. The use of light for brain stimulation is a disruptive advancement for both neuromodulation and DBS. This entirely different stimulation modality is brought about by a critical mass of innovations in molecular genetics and virology. Towne and Thompson (2016) define optogenetics as “*a method that uses light to control cells in living tissue, typically neurons, that have been modified to express light-sensitive ion channels and pumps*.” The foundation of optogenetics is a combination of genetic manipulations, which renders identified populations of neurons sensitive to the action of light. Development of optogentics would not have been possible without the research conducted on light sensitive algae by Nagel et al. (2002). The optogenetic approach was further championed by Deisseroth, Boyden, Miesenböck and Haegemann and has led to an explosive proliferation of different variants of light-sensitive ion channels, G protein-coupled receptors and ion pumps (Boyden et al., 2005; Lima and Miesenböck, 2005). In the last 8 years, optogenetics has become an established research tool for studying brain function. In fact, results obtained so far indicate that optogenetics provides unprecedented control and granularity of stimulation (Boyden et al., 2005).

So far optogenetics has been used predominantly as a research tool in animals, however applications in humans are not deemed impossible. There are patents in this direction filed by Boyden et al. (2012) and Deisseroth et al. (2015, 2016) to name but a few. Orphan status was recently granted to a viral-vector-based optogenetic therapy (from the company RetroSense Therapeutics) for retinitis pigmentosa, and initial clinical trials to evaluate safety are underway (Yun and Kwok, 2017). Microbial opsins do not possess toxic properties *per se*, therefore, applications in humans appear feasible from this perspective. Safety of the resulting therapy is, therefore, expected to depend mostly on the long-term properties of the genetic vector together with the safety of the implant. As such, safety aspects of the implants can be optimized to a sufficient extent based on the abundant experience with various types of DBS and other electrodes applied to neuromodulation and neuroprosthetics.

**Figure 1:**
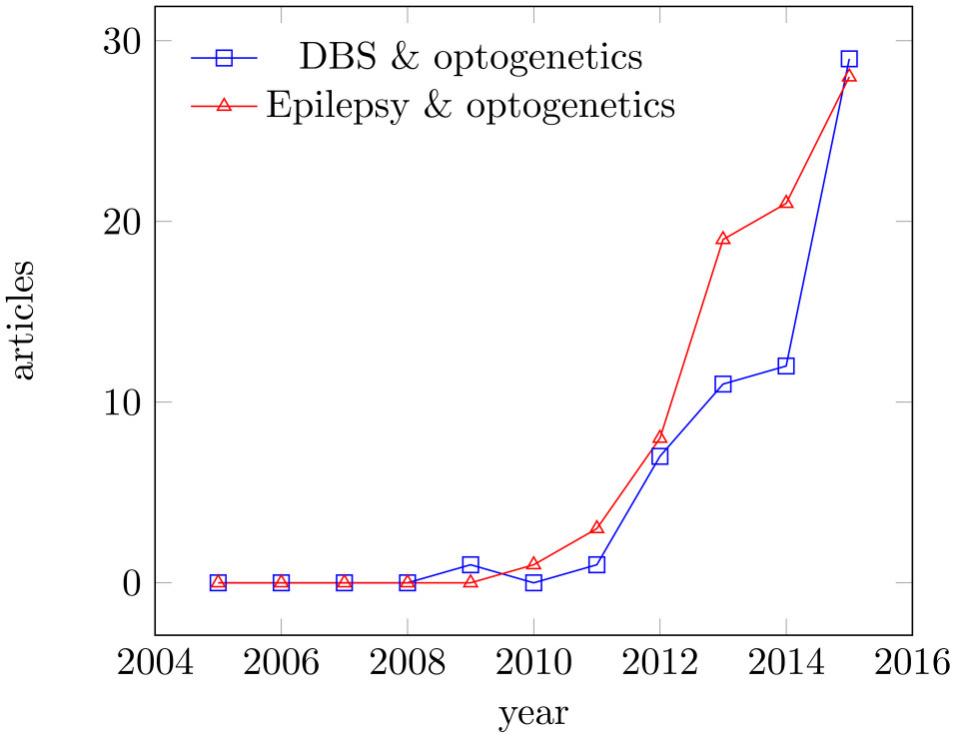
Articles published for the period 2005–2015. Articles published in Medline for the period 2005–2015; keywords: deep brain stimulation and optogenetics, epilepsy and optogenetics. Data were analyzed using the Medline trends tool (Corlan, 2004).

In this review, we will focus on *Parkinsons disease* with its local degenerative changes inducing a profound motor control disorder and *refractory epilepsy* as an example of a more distributed network disease, although often triggered by a focal lesion. Parkinson's disease and epilepsy can be taken ins some sense as extreme cases, since the acceptance criteria for eventual optogentics therapies can be very different. Both diseases get increasing attention in the literature as potential applications (see Figure 1). In Parkinson's disease, a loss of dopaminergic neurons leads to the loss of inhibitory gamma aminobutyric acid-sensitive input to the *subthalamic nucleus*. While there is no generally-accepted definition of refractory epilepsy (French, 2006), this term generally designates a spectrum of pathologies characterized by recurrent seizures, which respond poorly or not at all to conventional medicines. Clinical evidence indicates that some of these patients will actually benefit to some extent from add-on treatments while maintaining the antiepileptic drugs unchanged. At present, the main treatment options for refractory epilepsy are brain surgery (i.e., temporal lobe localized neocortical resection) and vagus nerve stimulation, which is a variety of neuromodulation (review in Cox et al., 2014). More recently, researchers started to explore possible optogenetic approaches as well (Wykes et al., 2016).

Neuromodulation, defined as as “the alteration—or modulation—of nerve activity by delivering electrical or pharmaceutical agents directly to a target area^2^,” will be considered in some alternative realizations. At present, clinically accepted neuromodulation and DBS therapies are still open-loop. However, the importance of simultaneous stimulation and recording from the neural tissue should not be overlooked. There are several reasons for this, including the possibility to better titrate the therapy and also to ensure appropriate timing of stimuli according to the state of the neural target. The sensing branches can control the stimulation regimen, adapt it to the state and needs of the neural system itself (Krook-Magnuson et al., 2013) or lock the activation under a given safety limit. In “controlled therapy,” sensing is essential to use physiological signals for triggering the therapeutic device (Zrenner et al., 2016). This is particularly important for epilepsy (Sorokin et al., 2017). Contrarily to electrical stimulation, optogenetics has the great advantage not to interfere with the recording of the tiny neural electrical signals. Already existing systems combine optogenetic activation with electric feedback loops (Emiliani et al., 2015; Yang et al., 2016).

The present review will not consider issues related to the optimizations and efficiency of light-sensitive ion channels and ion pumps, where there is a steady progress. Interested readers are directed to recent overviews by Wietek and Prigge (2016) and Deisseroth (2015) on opsin technology. Instead, we will address clearly unresolved issues, among which the biosafety of viral vectors.

## 2. A note on the mechanisms of DBS

The clinical use of DBS is mostly empirical, based on decades of experience with surgical ablative therapies. It must be emphasized that historically the major findings in both DBS and neuromodulation fields rest on serendipity and empirical observations because fundamental knowledge about the brain is still far from allowing complete understanding of patho-physiology as would be required to design optimal therapies. There are numerous historical examples ranging from the Renaissance era observations of Galvani to mid twentieth (Sironi, 2011) discovery of positive reinforcement by electrical stimulation (Olds and Milner, 1954).

It is an accepted clinical observation that both DBS (expected to stimulate the immediate target) and a lesion (silencing the same target) do alleviate symptoms of motor disorders despite their apparently opposite actions. In contrast, local stimulation leads to neuronal excitation and DBS affects symptoms on varying timescales and involving structures at different levels of organization.

To the present date, the mechanisms underlying the therapeutic effects of DBS remain insufficiently understood to predict applications in new therapeutic domain (recent reviews in Chiken and Nambu, 2016; McIntyre and Anderson, 2016). Even after 25 years of continuous application and research the circuit-level mechanisms of DBS remain elusive. While early hypotheses focused on the analogy between effects of ablation and those of stimulation, recent hypotheses on the mechanisms of DBS have shifted toward network-based theories, forgoing a direct link between lesioning and stimulation, and focusing instead on stimulation-induced disruption of pathological network oscillations (Johnson et al., 2008; McIntyre and Hahn, 2010; McIntyre and Anderson, 2016). It can even be conceived that DBS interferes with certain communication channels of the brain and thus disrupts pathological patterns of activity. The simplistic view of an imbalance between excitatory and inhibitory pathways must be complemented to take into account phenomena, such as retrograde activation, synchronicity in specific cells, interference with spontaneous (rhythmic) activity (Tass et al., 2012) and cell type activated (Witt et al., 2013). The role of astrocytes is well recognized but still difficult to integrate in our models (Kovacs and Pal, 2017). More and more, it is the limitations in physiological knowledge that impedes an optimal exploitation of techniques, such as optogenetics in the clinical world (Karas et al., 2013). On the other hand, optogenetics offers the new tools needed to further explore the brain and that is indeed what most application papers are about today (Gradinaru et al., 2009).

## 3. A multidisciplinary context

In this review, we argue that a number of independent technologies must converge to implement optogenetics as an effective therapy. We discern six major steps, which will be discussed in more details in subsequent sections:

1. The recombinant genetic technology required to develop a gene encoding the desired photosensitive ion-channel or any other desired protein.
2. The transfection technology required to introduce the new gene into target cells.
3. The genomic know-how needed to assure that the newly inserted gene gets activated.
4. The stereotaxic surgery which can play an essential role in the transfection by injecting agents into the appropriate target and by implanting the light stimulation device as required.
5. The engineering know-how of designing and producing an implantable optical stimulator is also significant and it represents a clearly distinct field of expertise, itself subdivided in several disciplines.
6. The clinical application of the optogenetic system involves the clinical and physiological knowledge, which is necessary to personalize parameter settings and optimize the clinical protocol.

Steps 1–3 are specific to optogenetics, while 4–6 are more generic.

It should be stressed that each technological domain listed above can also develop some forms of treatment on its own. Gene therapy (Collins and Thrasher, 2015) for example has been attempted long before optogenetics was even conceived. Direct insertion of functional proteins (Lin et al., 2017) or optic control of these proteins (Brechun et al., 2016) have also been suggested. The observation of Parkinson symptoms improvement after a localized brain lesion (Dubois et al., 1986) inspired the use of stereotactic brain surgery in patients. This old technique of stereotactic surgery (Lehman and Augustine, 2013) is facing a recent revival through the availability of new technological improvements (Starr et al., 1998). The effect of electrical stimulation used for anatomical guidance during ablative surgery finally resulted in the replacement of the brain lesion by a reversible DBS system (Benabid et al., 1996) and brain stimulators for human clinical use are now common. Hitherto, history of therapeutic progresses has very much resulted from serendipity and empirical findings rather than physiological knowledge based design. In addition to the need for more basic science, multi-disciplinarity characterizes modern brain therapies (Rossi et al., 2016). Genetics, pharmacology, photo-chemistry, cellular biology, surgery, neuroscience, optics, electronics, material science and various medical clinical specialties, to name but the most evident domains will have to collaborate. Unfortunately, there is often a significant tension between the required scientific freedom to transgress discipline boundaries on one hand and the institutional organization for teaching and employment of academics on the other hand (Osborne, 2015). Today, this constraint still represents a major challenge in a field, such as optogenetics, creating an urgent need for experts with overlapping skills and knowledge and, perhaps further thoughts about transdisciplinarity (Mittelstrass, 2011).

## 4. Light vs. electrical current

### 4.1. Electrical stimulation for DBS

Electrical activation of voltage sensitive channels is controlled by the voltage difference across the cell membrane, which drives ion currents. Sodium transient channel opening is activated by a membrane depolarization, thus by an inward current through the neural membrane. Other voltage sensitive channels have different responses to voltage and are more or less specific to other ions. The seven nanometer-thin lipid cell membranes form a relatively large electrical capacitance, requiring current to flow for some time before the activation threshold voltage can be reached. This explains the main part of the strength-duration relationship between activation threshold and stimulation pulse duration (Bostock, 1983). Assuming that the membrane conductivity is negligible compared to the extracellular space, Kirchoff's laws indicate that the current through the membrane is roughly proportional to the Laplacian (second spatial derivative) of the voltage along the membrane and a constant resistive factor. In peripheral nerves, the differential equations can be approximated by difference equations spanning over anatomically-defined distances determined by the nodes of Ranvier. These are considered as the only active spots along myelinated axons (Stephanova and Bostock, 1995). More complex volume conductor models have been developed for complex dendritic trees of the neurons in the central nervous system (Ranck, 1975). Not all identical neural structures are activated simultaneously but their recruitment depends on the anatomical position of the stimulation electrodes, the electrical properties (conductivity) of the intervening tissue and the anatomy of the neural structure itself. Variation of the waveform's shape can be used to improve the selectivity of stimulation in the peripheral nervous system by recruiting different groups of fibers. Readers are referred to the recent review of Grill et al. (2009) for update on the topic. Recent modeling studies point out some possibilities in the central nervous system in terms of neuromodulation by AC currents (Mahmud and Vassanelli, 2016).

Magnetic stimulation can be compared to electrical stimulation in the sense that the extracellular current generated by a pair of electrodes is now replaced by an induced current by a varying magnetic flux (Silva et al., 2007). In both cases, the extracellular current generates an extracellular potential field which induces the trans-membranous current. When that current has loaded the membrane capacitance up to a threshold potential, the voltage sensitive channels set up an all-or-nothing action potential.

Variables determining DBS outcome are the stimulation parameters and the positioning of the electrode. The stimulation parameters include the amplitude (current of voltage), frequency, and pulse width. These parameters play a role defining the volume of tissue activated (VTA) and in the therapeutic effectiveness of DBS (Butson et al., 2007). Typical clinically effective parameters for monopolar DBS are presented in Table 1.

**Table 1:** Typical clinically effective stimulation parameters in DBS.

**Voltage [V]**	**Pulse width [*****μs*****]**		**References**
1.3–4.4	60–120	130–160	Kuncel et al., 2006
2.2–3.6	60–90	130–185	Moro et al., 2002
1.0–3.0	60–120	130–185	O'Suilleabhain et al., 2003
1.0–3.5	60–210	100–185	Volkmann et al., 2002

*Typical monopolar DBS parameters used to treat patients can range from 1 to 4.4 V for the stimulation amplitude, 60 to 450 μs for the pulse width and from 90 to 185 Hz for the stimulation frequency*.

VTA in human subjects has been estimated in computational models (Maks et al., 2009). The more complete versions use patient specific imaging data that enrich the models with anatomical information. They also incorporate electrical characteristics, such as impedance and anisotropy, of the different brain parts surrounding the electrode. The study by Maks et al. (2009), found volumes varying from 30 to 116 mm^3^ with an average of 71 mm^3^ (i.e., 5 mm diameter). The results from this study further suggest that smaller stimulation volumes in the subthalamic nucleus (STN) are better at alleviating Parkinson's symptoms. The model was experimentally verified indirectly by estimating the stimulation volume spreading into the *capsula interna* for different voltages and comparing this to muscle twitching of the arm or leg by patient stimulation of the *corticospinal tract* (Butson et al., 2007). Other studies from this laboratory measured the voltage spread in the brain of a rhesus monkey and compared it to the one predicted by the model (Miocinovic et al., 2009). The study found that the model accurately predicted the voltage distribution observed *in vivo*.

### 4.2. Light stimulation and opsins

Within the tissue, photons interact with biological matter via various processes, which can be broadly categorized into scattering and absorption. An accessible overview on current photonics medical applications is given in Yun and Kwok (2017). Light works in a different way upstream of the activation of the membrane ion channels. Here, it is the optical properties of light across the tissues that will determine the threshold and target selectivity. In the case of the well-studied ion channel ChR2, the blue light will cause all-*trans*-retinal to isomerize to 13-*cis*-retinal, and so a conformational change in the protein will open the ChR2 channel to allow cations to enter and depolarize the cell. Switching the light off again will cause the 13-*cis*-retinal to revert to its original state closing the channel and thereby repolarizing the cell. These ion channels do not close spontaneously (as the voltage sensitive sodium channel does) and the resulting response dynamics is in the first place dependent on the opto-chemical properties and density of the photosensitive channels. The strength-duration relationship or the significance of the stimulus duration is now entirely different despite still linked to electrical charges on the membrane capacitance. When open, these charges depolarize the membrane by providing a leakage current. Only then the membrane capacitance starts loading and the density of the available channels is thus much more a limiting factor. The strength-duration relationship still corresponds to a membrane capacitance being loaded, but no longer through the same current.

It should be noted that even if the stimulus does not produce an action potential, a membrane potential shift occurs, such that very long stimulus pulses will modulate spontaneous activity. New phenomena can thus be expected to occur (Grossman et al., 2011). For example, on striatal brain slices, dopamine release (which is an effect pursued in treating Parkinson's disease) appears to be stable when electrically stimulated while a rundown effect is observed with optical stimulation (O'Neill et al., 2017). Once the membrane depolarization voltage has reached the threshold value, however, the same voltage-controlled mechanism as described for electrical stimulation will launch a propagated action potential. Activation selectivity still depends on the geometry and intervening tissues. However, whereas electrical stimulation is controlled by the electrode position, the membrane ion channel distribution and roughly constant resistivity, here, source orientation, source optic features (color, polarization), optic characteristics of intervening tissues, channel density and opto-sensitivity, are the key parameters defining the threshold, selectivity and recruitment.

Selectivity also arises from the use of cell-specific promoters, which drives the expression of the protein in the cells. The opsin is placed downstream of a strong promoter such as synapsin, CMV or CAG. This will lead to strong expression of the opsin in almost all of the cells where the construct is present. Alternatively, one can opt to target cell-specific promoters such as alpha-calcium/calmodulin-dependent kinase II (α-CamKII), which is expressed in forebrain pyramidal neurons. Where electrical stimuli can in some conditions block the propagation of action potentials, similar effects are now obtained with infra-red pulses (Walsh et al., 2016). Some opto-sensitive channels can work as cell silencers, which is not the same as the selective activation of inhibitory pathways, which both techniques could in principle achieve (Malyshev et al., 2017). Through color selectivity, a single optic device could now selectively inhibit or activate the same cells.

Geometric or anatomical selectivity is in principle available to optic stimulation as well as to electrical micro-electrodes (McCreery et al., 2006). Activating light oriented toward a specific points is a realistic goal in examples, such as the retinal prosthesis (Soltan et al., 2017). Optical techniques are being developed that could achieve similar or better selectivity and parallelism than the electrical equivalent (McAlinden et al., 2015; Conti et al., 2016). It should be emphasized that the placement of electrodes is entirely dependent on the surgery. Electrode placement is also a selectivity factor in the frame of optogenetics but selective transfection is another possibility so that only the targets are made photo-sensitive. Selective activation of specific cells, as can be achieved in the frame of optogenetics, could become a key to a successful therapy (Yekhlef et al., 2017). However, the impact on the therapeutic effect cannot yet be established on the basis of the rather preliminary information available today.

### 4.3. Functionality

The architecture of the brain nuclei poses a problem for purely electrical stimulation because electrodes are relatively indiscriminate with regards to the underlying physiology of the neurons that they stimulate. Typically, physical proximity of the stimulating electrode contact to the neuron is often the determining factor as to which neurons will be stimulated (Deisseroth et al., 2014). Accordingly, it is not considered feasible to restrict stimulation to a single class of neurons with electrical stimulation while optogenetics promises to do just that and even to activate specific channels within the same neurons.

In optogenetics, the volume of tissue that gets activated is also determined by the interaction of several processes. Neuronal activation depends on the absolute amount of light that reaches the neurons, the efficiency of the transfection and the sensitivity of the opsin. Besides these, neuronal physiological properties and the network in which the neuron is embedded also influence how effectively light can control the activity. The propagation of light in the brain is determined by the light absorption and scattering (Vo-Dinh, 2003). How far light reaches inside the brain can be estimated using Monte Carlo simulations. Different studies have calculated that the light intensity drops to 1% at a distance 1 mm away from the emission point (Bernstein et al., 2008; Chow et al., 2010; Stujenske et al., 2015). Additionally, these simulations show that the light distribution inside the brain has an ellipsoidal shape depending on the wavelength, the size and type of light source and the emission aperture angle (i.e., numerical aperture). The simulations also show that a portion of the light back scatters and illuminates the tissue behind the optical fiber. This effect gets exacerbated at higher emission powers to the point that, in some cases, the tip of the fiber is at the center of the illuminated volume. Regarding the sensitivity, studies in acute slices and *in vitro* show that maximal channel activation for different channelrhodopsin 2 (ChR2) variants is achieved at a power density of 10 mW/mm^2^, with only 50% of the channels getting activated at 1 mW/mm^2^ (Wang et al., 2007; Lin et al., 2009). If an optgenetic DBS would deliver light through an optical fiber with a core diameter of 200 μm and a numerical aperture of 0.22, the data obtained by (Stujenske et al., 2015) can be used to estimate the power required to achieve an activation volume similar to electrical DBS (i.e., 5 mm sphere). For maximal neuronal acativation, a total of 1.5 W would be required to obtain a power density larger than 10 mW/mm^2^ within the illumination volume. Assuming that the tip is indeed at the center. The required power gets exponentially higher if it is not. If the criteria are relaxed and only a power density of 1 mW/mm^2^ is provided at the end of the volume only 150 mW should be delivered. The first value is prohibitively high and would probably create some direct damage in the brain. The second is also quite high, although the brain might be able to withstand it. However, a portable device that has to deliver that amount of power would probably not be practical. Tapered fibers have been tested recently by Pisanello et al. (2017) who showed that varying tapering angle allows for obtaining selectivity of stimulation in vertical direction. Authors claim that achieving uniform effective illumination of large brain structures with minimal invasiveness and light power. In addition, tapering permits smooth insertion into the brain, which also minimizes tissue reaction.

So-stated arguments indicate that stimulation paradigm with ChR2 analogous to DBS might not be the best approach with common ChR2 variants. An alternative could be to use recent variants like ChR2-XXL, which is 10,000 times more sensitive with the disadvantage that it is slower (Dawydow et al., 2014). However, this approach does not take advantage of the capabilities of the optogenetical tools for specific cell type stimulation. Selectivity of stimulation has been tested in rodent models with some positive results (Gradinaru et al., 2009). As an alternative, the inhibition modality of optogenetics could be used. A widely accepted hypothesis of DBS for Parkinson's disease is that excitatory neurons in the STN are inhibited (Shin et al., 2007; Sutton et al., 2013). This hypothesis has been tested by two different groups in rodent models of Parksinosn's disease (Gradinaru et al., 2009; Yoon et al., 2014, 2016). Unfortunately, the results obtained by each group contradict the other, although Yoon et al. (2014) attribute the difference to the animal model and the test used to evaluate Parksinosn's disease improvement by the other group. Tables 2, 3 show the efficacy of applying optogenetic stimulation or inhibition to rodent models in different studies. The tables have a line-to-line correspondence to facilitate comparison. Although, there is yet no definitive conclusion regarding the use of optogenetics of the treatment of Parkinson's disease, these studies demonstrate that optogenetics would be an indispensable tool to better understand mechanisms of Parksinosn's disease and DBS.

**Table 2:** Summary of the efficacy of applying optogenetics in Parksinosn's disease rodent models, I.

**Cell type**	**Wavelength**	**Opsin**	**Frequency (Hz)**	**References**
Excitatory glutamatergic	561	eNpHR	I	Gradinaru et al., 2009
Astroglia	473	ChR2	I	Gradinaru et al., 2009
Excitatory glutamatergic	473	ChR2	130	Gradinaru et al., 2009
Excitatory glutamatergic	473	ChR2	30	Gradinaru et al., 2009
Afferent axons	473	ChR2	130	Gradinaru et al., 2009
Afferent axons	473	ChR2	20	Gradinaru et al., 2009
Projection neurons	473	ChR2	130	Gradinaru et al., 2009
Projection neurons	473	ChR2	20	Gradinaru et al., 2009
Excitatory glutamatergic	590	NpHR	I	Yoon et al., 2014
Excitatory glutamatergic	590	NpHR	I	Yoon et al., 2014
Excitatory glutamatergic	590	NpHR	I	Yoon et al., 2014
Excitatory glutamatergic	590	NpHR	5	Yoon et al., 2016
Medium spiny neurons	473	hChR2(H134R)	CW	Hernández et al., 2017

*I, intermittent stimulation*.

**Table 3:** Summary of the efficacy of applying optogenetics in Parksinosn's disease rodent models, II.

**Cell type**	**Effect**	**Model**	**Test**	**Success**	**References**
Excitatory glutamatergic	Inhibition	Rat, 6-OHDA	Amphetamine/Rotation	No	Gradinaru et al., 2009
Astroglia	Inhibition	Rat, 6-OHDA	Amphetamine/Rotation	No	Gradinaru et al., 2009
Excitatory glutamatergic	Activation	Rat, 6-OHDA	Amphetamine/Rotation	No	Gradinaru et al., 2009
Excitatory glutamatergic	Activation	Rat, 6-OHDA	Amphetamine/Rotation	No	Gradinaru et al., 2009
Afferent axons	Inhibition	Mouse, 6-OHDA	Amphetamine/Rotation	Yes	Gradinaru et al., 2009
Afferent axons	Inhibition	Mouse, 6-OHDA	Amphetamine/Rotation	Worsen	Gradinaru et al., 2009
Projection neurons	Stimulation	Mouse, 6-OHDA	Amphetamine/Rotation	Yes	Gradinaru et al., 2009
Projection neurons	Stimulation	Mouse, 6-OHDA	Amphetamine/Rotation	No	Gradinaru et al., 2009
Excitatory glutamatergic	Inhibition	Rat, 6-OHDA	Stepping	Yes	Yoon et al., 2014
Excitatory glutamatergic	Inhibition	Rat, 6-OHDA	Cylinder	No	Yoon et al., 2014
Excitatory glutamatergic	Inhibition	Rat, 6-OHDA	Apomorphine/Rotation	No	Yoon et al., 2014
Excitatory glutamatergic	Inhibition	Rat, 6-OHDA	Apomorphine/Rotation	Yes	Yoon et al., 2016
Medium spiny neurons	Stimulation	Rat, 6-OHDA	stereotypic behavior	Yes	Hernández et al., 2017

*The studies used different power settings: Gradinaru et al. (2009) used 10/200 μm fiber core; Yoon et al. (2014, 2016) used 1.1/200 μm core fiber; Hernández et al. (2017) used 10 mW output power*.

We have seen that electrical currents and light do not necessarily activate the same structures. In addition, photosensitive channels have been developed for specific ions and superposition of different light wavelengths allows for activation/inhibition combinations that are not possible with electrical stimuli. However, once action potentials are launched, they can no longer be distinguished on the basis of the triggering event. Parameters, such as pulse frequency, train duration, train rate, treatment session duration, duration of the therapy, event-triggering of the therapy and so on are expected to yield the same effects if the same structures were activated and, this is where a serious word of caution is warranted. In addition, the neural system is an adaptive network. Plasticity, as a general phenomenon can completely modify long-term effects.

Long-term consideration is thus not only important to check the lifetime of implanted device or to evaluate unwanted side effects but also to ensure effectiveness of the treatment strategy (Jarvis and Schultz, 2015). As “accelerated aging” methods do not work here, chronic studies and high sensitivity detection seem to be the only alternative.

## 5. Optogenetics

At present, optogenetic ion channels are designed routinely on the basis of the family of the bacterial opsins. In hindsight is seems surprising that applications in the neural system took as long as 40 years after the initial isolation of the halobacterial rhodopsin. Only, by 2010, the major classes of ion-conducting microbial opsins have been demonstrated to be able to excite neurons (Yizhar et al., 2011). Optogenetic proteins can be classified into several overlapping groups by the mode of their actions (i) fast-inhibiting; (ii) fast exciting; (iii) step function opsins (i.e., bi-stable); (iv) modulated biochemically (review in Yizhar et al., 2011). A large variety of channels with different absorption spectra and kinetics have been designed. The temporal precision based on the use of custom opsin designs offers unprecedented opportunity for modulation of defined neuronal populations. Such perspective is in a sharp contrast with the relatively “broad–band” electrical stimulation traditionally employed in DBS.

### 5.1. Variants of optogenetic approaches

#### 5.1.1. Overwhelming possibilities

In addition to forms sensitive to different wavelengths, the classical opsins such as DNA/channelrhodopsin 2 (ChR2) have been complemented with Halorhodopsin (NpHR) that hyperpolarizes the Cl- pump (Klapper et al., 2016) and Archaerhodopsin-3 (Arch) that forms a light sensitive proton pump (Mantoan Ritter et al., 2014). As already mentioned, combining these makes it possible to control different effects using different light colors (Stark et al., 2012). More recently, several cellular control methods have been developed around the possibility to modulate G-proteins (Kleinlogel, 2016) thus opening a new branch of optogenetics with new applications in perspective. Alternatively, genes for endo-cellular molecules (for example genes expressing fluorescent proteins for bio-sensing Enterina et al., 2015) as well as various ion channels can be integrated in the cell genome. For example, intracellular calcium can be specifically controlled (Mager et al., 2017). Key chemicals controlling cellular biology (precursors, enzymes, neurotransmitters and their agonists, sensors or signaling molecules (Smedemark-Margulies and Trapani, 2013), can be delivered to the brain in an inactive form (drug-encapsulating liposomes, caged molecules or photosensitive inactivating bounds) and activated under light control (Nakano et al., 2016). In other applications, optochemistry uses photosensitive uncaging (glutamate release in acute experiments) (Venkataramani et al., 2007). There are also examples of photoreceptors that can be used as “optogenetic switches” (Salinas et al., 2017). Astrocytes can be selectively activated by light-activated Gq protein-coupled opsins (Mantoan Ritter et al., 2014). Among the non-neuronal cells that can be activated, oligodendrocytes might also become important therapeutic targets (Lee et al., 2016). The new possibility to modify synapses (Sinnen et al., 2017) could become a major tool in correcting or adjusting neural pathway balances. Even unexpected possibilities, such as controlling cell mechanical interactions, might become possible (Valon et al., 2017).

#### 5.1.2. Limitations

As a rule, the introduced foreign proteins trigger a foreign body immune reaction. In addition, proteins tend to be catabolized so that only gene modifications can enable a chronic effect. Direct introduction of functional proteins seems limited to acute experiments.

#### 5.1.3. Possible applications

All monogenic diseases are in principle candidates for gene therapy. Many clinical trials have already taken place in various diseases outside the neural system: myopathies, haemophilia, retinitis pigmentosa are just a few examples (Collins and Thrasher, 2015). Genetically encoded sensors have become essential research tools and might later offer perspectives for “closed loop systems” (Emiliani et al., 2015; Yun and Kwok, 2017). Bioluminescent indicators for voltage and various ion sensors are commercially available (Inagaki et al., 2017).

## 6. Viral vectors

From the point of view of biotechnology, viruses are almost perfectly evolved nano-machines for gene delivery. Viruses can infect host cells and completely overtake their metabolism reprogramming it to serve only for their replication. Outside the host cell, the nucleic acid (i.e., DNA or RNA) forming the genome is encapsulated in a protected shell called *capsid* forming a particle with dimensions in the range of 20–200 nm. The particle itself is called *virion*. The proteins on the surface of the capsid are responsible for the target specificity and incorporation in the cell.

Rapid development of viral vector biotechnologies allows for selective modification of the wild type virus properties. Viral genomes can be edited and the genes, encoding pathogenic functions, can be removed and replaced by different genes allowing for engineering of the target cell's function. Such engineered construct is called a *viral vector* and it has important differences by design compared to the native prototype (i.e., the wild type virus).

- The most important difference is that by design, viral vectors do not replicate. This feature is achieved by deliberately removing all replication-specific genes from the prototype viral genome. This has impact on the production of the vector. Often the viral genome is segregated into 2–4 different plasmids.
- Viral vectors retain their invasiveness. They can infect host cells and inject their genetic material in the cytoplasm and in some cases integrate in the eukaryotic genome.
- Viral vectors are selected for their specificity.

Many viruses have been used as prototypes of viral vectors, however only few types are considered suitable for human application. Readers are directed to the recent reviews of Gray et al. (2010b), Lentz et al. (2012), and Kantor et al. (2014) for specific gene-therapy and production aspects of viral vectors. Since the envisioned genetic manipulation making the brain susceptible for optogenetics aims to treat specific diseases one has to consider it as a type of gene therapy. Therefore, all safety and ethical properties of such therapies should apply in further analysis. We give an initial consideration of the issues in the subsequent sections.

### 6.1. Safety of gene therapy and viral vectors

Gene transfer experiments in humans started in 1990s but were hampered by initial failures and serious adverse effects, which occurred in the early trials. By present, viral vector technology advanced substantially because of the substantial improvement of our understanding of how different viruses interact with the organism and how their genomes can be designed to reduce biohazard and improve efficiency.

### 6.2. Adenovirus vectors

Historically, the first applications of gene therapy used adenoviruses and retroviruses. An adenovirus is a non-enveloped particle of size ranging between 70 and 100 nm. Adenoviruses (AV) have been isolated from a large number of species and tissue types, and in humans, they cause mild respiratory illnesses and gastroenteritis. The virus genome consists of linear double stranded DNA of approximately 36 kbp. An AV particle enters a cell via receptor-mediated endocytosis. The virus escapes the endosome and translocates to the nucleus where it delivers its DNA, which stays *episomal*. AV can efficiently infect a wide variety of cell types independent of the phase of the cell cycle (Harui et al., 1999).

While adenovirus vectors are very efficient at delivering genes, adenovirus vectors can cause toxic effects that limit their efficiency and safety. AV are highly immunogenic. Upon contact with the virus, the human immune system mounts a full-scale assault, including CD4+ T-helper cells, CD8+ cytotoxic-T cells, and NK cells, in order to clear the virus (Xu et al., 2010). Intravenous AV vector delivery for gene transfer purposes, especially at high doses, stimulates strong innate and adaptive immune responses and can be fatal for the host as notoriously found out in the first clinical trials. Further deletions of the adenoviral genes have yielded helper-dependent adenovirus (HD-Ads) or “gut-less” vectors (Kochanek et al., 2001), which are completely devoid of viral protein coding sequences. This has decreased immunogenicity and also prolonged transgene expression.

In systemic application AV are sequestrated by the liver Kupffer cells and cause toxic effects there. AV vectors also activate the complement system (Manickan et al., 2006; Tian et al., 2009). In addition, high doses of adenovirus vectors rapidly induce a burst of platelet activating factor *in vivo* that can lead to shock.

From this overview it can be concluded that AV vectors are sub-optimal for gene delivery in the brain due to their immunogenicity (i.e., possibility ot trigger gliosis and neurodegeneration) and low specificity.

### 6.3. Adeno associated virus (AAV) based vectors

AAV forms small, non-enveloped virions with icosahedral symmetry. AAV belongs to the Parvoviridae family and has single-stranded DNA (see Table 5). Multiple serotypes of AAV have been described (AAV1–AAV9) with distinct tissue tropism and transduction efficiency (see Table 4). AV can not replicate by itself and is not related to any known human pathology. AAV can replicate only in co-infection with helper viruses, such as AV, HSV or papiloma virus (HPV) (Weitzman and Linden, 2011). For example, the adenovirus acts as a helper virus by supplying the E1a, E1b, E2a, E4orf6 and viral-associated RNA genes.

**Table 4:** Some properties of AAV serotypes.

**Serotype**	**Receptors**	**References**
AAV1	N-linked α2, 3/α2, 6-Sialic acid	Wu et al., 2006; Ng et al., 2010
AAV2	Heparan sulfate, Integrins α/β5/α5β1, FGFR1, HGFR, laminin receptor	Summerford and Samulski, 1998; Qing et al., 1999; Akache et al., 2006
AAV3	Heparan sulfate, FGFR1, HGFR, laminin receptor	Rabinowitz et al., 2002; Akache et al., 2006; Blackburn et al., 2006; Ling et al., 2010
AAV4	O-linked α2, 3-Sialic acid	Kaludov et al., 2001
AAV5	N-linked α2, 3-Sialic acid, PDGFR	Kaludov et al., 2001; Walters et al., 2001; Di Pasquale et al., 2003
AAV6	N-linked α2, 3/α2, 6-Sialic acid, heparan sulfate, EGFR	Wu et al., 2006; Ng et al., 2010; Weller et al., 2010
AAV7	Unknown	
AAV8	Laminin receptor	Akache et al., 2006
AAV9	N-linked β1, 4-Galactose, Laminin receptor	Akache et al., 2006; Shen et al., 2011

*The table is based on Kantor et al. (2014). FGFR, fibroblast growth factor receptor; HGFR, hepatocyte growth factor receptor; PDGFR, platelet-derived growth factor receptor; EGFR, epidermal growth factor receptor*.

**Table 5:** Virus-derived characteristics.

	**Adenovirus (AV)**	**Lentivirus**	**AAV**	**HSV**
Particle size	70–90 nm	80–120 nm	18–26 nm	120–300 nm
Genome size	37.7 kbp	9.7 kbp	4.7 kbp	150 kbp
Nucleic acid type	DNA	RNA	DNA	DNA
Genome structure	ds linear	ss linear (+)	ss linear (+/−)	ds linear
Envelope	None	VSVG glycoprotein	None	Glycoproteins

*ss, Single strand; ds, double strand*.

The AAV genome consists of two 145 base-pair inverted terminal repeats (ITR), *rep* and *cap* genes. The ITRs form loop structures and are the only cis-acting elements that are necessary for genome replication, integration and packaging in the capsid (Kantor et al., 2014). The wild type AAV genome encodes four rep proteins—Rep 78, 68, 52, and 40; three viral structure proteins forming the capsid—VP1, 2 and 3; assembly activating protein, which is involved in the translocation to the nucleolus—AAP (review in Smith, 2008).

The capsid determines the tissue specificity or tropism of a given virus by regulating the immediate cellular response to the virus, mediating pathways for internalization into the cell, and functions in the uncoating process within the nucleus. Specific regions of the capsid proteins interact with receptors and co-receptors on the host cellular surface to mediate the viral infection process and serotypes can differ with respect to the receptors that they bind to. AAV infects a host cell through receptor-mediated endocytosis.

Approximately 80% of the population is seropositive for anti-AAV antibodies (review in Weitzman and Linden, 2011). Also approximately 60% of the population has neutralizing antibodies at age 10, which persists into adulthood. Nevertheless, surprisingly little is known about the life cycle of AAV in humans.

The AAV vectors can stably transfect tissues in different species, including humans. Reports demonstrate durations of such tranfections of at least 6 years in primates (Rivera et al., 2005), 8 years in dogs (Niemeyer et al., 2009) and more that 10 years in the human brain (Leone et al., 2012).

AAV is unique among mammalian viruses in that it integrates into a distinct region of the human chromosome 19, the so called AAV integration site AAVS1 (review in Smith, 2008). The site-specific integration of AAV requires the presence of two viral elements: the inverted terminal repeats (ITRs) and nonstructural proteins Rep78/68. Accordingly, all current AAV vectors lacking the *rep* gene lack the capacity for site-specific integration. Wild-type AAV sequences are integrated into the host cell genome as tandem, head-to-tail repeats linked to genomic DNA sequences by the viral inverted terminal repeat elements (or ITRs). Specificity is achieved through the interaction of a glycine-rich loop that binds the major groove and an α-helix that interacts with a downstream minor groove on the same face of the DNA. In contrast, the integration of recombinant AAV genomic sequences in the absence of the AAV Rep proteins is inefficient and is not limited to chromosome 19. It is estimated that only about 10% of recombinant AAV sequences integrate into the host cell genome, thus indicating that the majority of vector genomes persist in an extrachromosomal form *in vivo* (Smith, 2008).

### 6.4. Herpes simplex virus (HSV) derived vectors

HSV is a member of the *Herpesviridae* family. There are two main viral types—HSV-1 and HSV-2. While HSV-1 causes orolabial lesions and resides in the trigeminal ganglion, HSV-2 causes genital lesions and resides in the sacral ganglia. About 40% of the adult population is seropositive for HSV-1 in the developed countries (review in Kantor et al., 2014). The HSV particles have icosahedral capsid covered by a lipid bilayer envelope. The envelope includes glycoproteins, which are essential for the viral entry into the cell. The viral genome consists of dsDNA of 152 kb (see Table 5). The life cycle of HSV is particular: the cycle has two alternative phases—lytic and latent. The lytic pathway leads to viral proliferation, emission and imminent cell death, while the latent pathway causes the virus to form an episomal particle within the nucleus in a dormant state. The latent viral particle is capable of lytic transformation upon the action of physical factors (i.e., cold, heat shock etc.). Viral replication is a multistage process, controlled by many genes, which can be removed from the genetic backbone of the vector. This ensures large packaging capacity of up to 125 kbp. What makes HSV suitable for CNS and PNS applications are the following properties: (i) the wild type virus propagates trans-synaptically in both anterograde and retrograde directions; (ii) the wild type virus is neurotropic and (iii) stability of the latent phase.

Infection with HSV typically occurs at the cutaneous or mucosal epithelium where replication of the virus is initially lytic. The virus can also invade axons of sensory neurons in the affected area and undergo retrograde transport to the dorsal root ganglia where it can switch to a latent phase.

Once the virus has reached the nucleus of a cell, the linear genome circularizes and is maintained as an episome with minimal integration (Lentz et al., 2012). HSV vectors demonstrate extensive host cell range, high efficiency gene transfer, and enhanced safety, as persistence of the genome as an episome decreases the likelihood of insertional mutagenesis (Shayakhmetov et al., 2010). Due to cytotoxicity associated with viral gene expression non-replicating and *amplicon* vectors have been developed. HSV amplicons are eukaryotic expression vectors that harbor the HSV origin of replication and cleavage/packaging signals. HSVs do not require pseudotyping to increase neurotropism, as these viruses are inherently neurotropic. HSV-mediated gene expression has a rapid onset (< 1 day) (Penrod et al., 2015). Transgene expression mediated by HSV vectors has been demonstrated to persist for up to 7 months in the rat brain, but may not be stable (Zhang et al., 2000; Sun et al., 2003).

Replication-attenuated and replication-deficient HSV vectors have the transgene of interest inserted into the viral genome, with targeted deletion of specific immediate early (IE) genes to disable the lytic cycle and render these vectors non-toxic (Wu et al., 1996; Krisky et al., 1998).

### 6.5. Lentriviral vectors

Lentivirial vectors are derived from the HIV genome and there is abundant literature about their properties and safety. Interested readers can consult the recent review of Kantor et al. (2014). Lentriviral vectors lead to permanent genetic modification, which may not be desirable in all cases. Since neurons in the majority of brain areas do not divide, there is a little justification, from medical ethics perspective, why a permanent genetic modification of the patient's brain tissue is desirable.

### 6.6. Gene therapy in CNS related to the applications

The CNS has proven quite permissive to viral vector gene transfer and expression for many of the conventional delivery vectors. AAV, lentiviral, and to a lesser extent HSV vectors, are the most frequently utilized agents for brain and spinal cord gene delivery. At present, AAV vectors are the leading platform for gene delivery in CNS. Beyond the large number of preclinical and basic mammalian studies involving AAV delivery to the brain and/or spinal cord, the large majority (approximately 75%) of clinical trials that have been initiated for CNS gene therapy have utilized AAV, as contrasted to trials utilizing adenovirus (6%), lentivirus (12%) or retrovirus (6%) (Gray et al., 2010a).

In a recent clinical trial, 12 patients with advanced Parkinson's disease had an application of an AAV vector carrying a transgene encoding glutamic acid decarboxylase (GAD) (Kaplitt et al., 2007). The therapy was well tolerated, with no adverse effects attributable to gene therapy noted for any of the patients. Observed improvement in motor activity lasted for at least 1 year. The trial using AAV-GAD did not establish adverse effects for up to 12 months (Feigin et al., 2007). Another trial for Parkinson's disease using AAV-hAADC vector for dopamine replacement established favorable safety profile (*n* = 5 patients) but low efficacy (Christine et al., 2009). Similar safety profiles have been established in another trial (Muramatsu, 2010). A phase 2 study using 45 patients and AAV-GAD did not establish serious adverse affects attributed to the treatment (LeWitt et al., 2011). So-reported results demonstrate that AAV is a sufficiently safe vector for applications in the brain. In summary, the recombinant AAV vectors have emerged as a viable delivery method for human gene therapy as they can be designed to meet the precise treatment needs of a given disease by delivering a gene to specific cell types within the affected tissues with a minimal immune response.

### 6.7. Assessing the risks of the genetic modification

Genetic modification has some inherent risks. A comprehensive treatment of the principles of risk assessment will require a dedicated publication. Interested readers can consult Baldo et al. (2013) for treatment of gene therapy cases. Briefly, the methodology of risk assessment consists of the following steps:

- hazard identification;
- hazard characterization;
- risk estimation, i.e., risk band estimation;
- evaluation of risk management options based on the assigned risk band.

Considering viral vector applications the following principal hazards can be identified:

- Immune system reaction
- Pleiotropic effects due to low specificity
- Recombination of the vector (older generation vectors)
- Insertional mutagenesis and carcinogenesis (older generation retroviral vectors)

Various candidate viral vectors are compared in Table 5, based on the studies of Doherty et al. (2011) and Howarth et al. (2010).

Transfection-related properties of different vectors are summarized in Table 6.

**Table 6:** Transfection properties of viral vectors most common in human gene therapy.

	**Adenovirus (AV)**	**Lentivirus**	**AAV**	**HSV**
Payload	3.0–8.0 kbp	2.5–8.0 kbp	2.5–5.0 kbp	16.5^*^–125 kbp
Latency to peak transgene expression	3–5 days	7 days	2–4 weeks	3–5 days
Rate limiting step before expression	Translocation to nucleus	Genome integration	Second strand synthesis	Translocation to nucleus
Integrates in host genome	No, but in nucleus	Yes, non-specific	Yes, inefficient	No
Expression requires integration?	No	Yes	No	No
Transduces post-mitotic cells?	Yes	Yes	Yes	Yes
Duration of transgene expression	Weeks/months	Years	Years	Weeks/months

* *Miyagawa et al. (2015) but up to 120 kbp theoretically (Kantor et al., 2014); bp, base pair*.

We can identify some desirable properties of the ideal vector, considering the specific application:

- very low insertional mutagenesis potential—due to necessity of long-term action;
- very low immunogenic potential—for the same reasons and considering the importance of chronic neuroinflammation in the pathogenesis of neurodegenerative diseases;
- neurotropism;
- low recombination potential.

The payload capacity is not a differentiating factor for optogenetics applications as the channelrhodopsins are in the range 1.7 kbp (i.e., hChR2-GFP). Based on these criteria, a preference table can be assembled (Table 7). From the table it appears that, given the present state of development of the viral vector technology, the best choice for optogenetic application in human is AAV followed by HSV.

**Table 7:** Summary of viral vector comparison.

	**Adenovirus**	**Lentivirus**	**AAV**	**HSV**
Insertional mutagenesis potential	Very low	Moderate	Very low	Very low
Immunogenicity	Moderate	Very low	Very low	Moderate
Neuronal transduction	moderate	Moderate	Moderate	Strong
Glial transduction	Strong	Moderate	Low	Low
Permanent effect	No	Yes	No	No
Duration of expression	Long	Long	Long	Short
Recombination potential	Low	Very Low	Low	Low
Preference	4	3	1	2

### 6.8. Alternatives for gene delivery

Non-viral alternatives for gene delivery have been developed in parallel to viral vector technology. These are for example, transfection by electroporation (Nomura et al., 2016) or focused ultrasounds (Wang et al., 2017). Another potential alternative is laser photoporation, currently developed *in vitro* (Antkowiak et al., 2013). For various reasons however, it is the viral vector-based gene delivery that stands out as the most mature transfection technology.

## 7. Implanted optical brain stimulator

Research on implantable hybrid optoelectronic probes has progressed rapidly in the last couple of years. Optical stimulation can be applied in several ways. Available techniques include fiber optics, on-probe μLED-s and waveguide-based approaches. A recent overview on the topic can be found in Iseri and Kuzum (2017). Focal optic stimulation can be achieved with proper optical design, for example using independently controllable GaN light emitting diodes (McAlinden et al., 2015). Optical brain stimulators can work with several wavelengths to activate selectively differently transfected cells within the same region (Emiliani et al., 2015); or they can be designed to provide complex illumination patterns (Segev et al., 2017).

### 7.1. Alternative realization

Micro and perhaps nanotechnologies are at stake here when considering the development of the appropriate stimulation system (Pisanello et al., 2016). Multi-functional devices can combine photo-stimulation and electrical recording (Wang et al., 2012) including micro-electrode arrays placed in the vicinity of the target (Buzsaki et al., 2015; Naughton et al., 2016).

### 7.2. Limitations

Designing probes that can be safely inserted in the brain is a significant challenge. Generic tissue reactions to the implant device have been discussed elsewhere (Prodanov and Delbeke, 2016a). For any implanted device, power dissipation and thus local temperature must be kept at an acceptable level (Arias-Gil et al., 2016; Shin et al., 2016). The strong light power used in photogenetic could transform photochemically some cell molecules into toxic agents. Chronic aspects are important to consider: a good example is the mitochondria-mediated apoptosis induced by prolonged ChR2 activation (Perny et al., 2016). Whether electric, microfluidic or optical, any application must take into account the diffusion and depth of penetration. Although only mentioned in animals, light leaks might influence an implant (Eckmier et al., 2016), considering that near infra-red spectroscopy is a technique based on light transmission through scalp, skull and brain (Benaron et al., 2000). Optic implants are new and require a specific encapsulation still lacking chronic evaluation (Rossi et al., 2015). On the other hand, it is much easier to make optic devices Magnetic Resonance Imaging (MRI) compatible than their electrical counterparts. This is an important factor for the modern clinical practice.

### 7.3. Possible applications

DBS is presently used in Parkinson's disease, epilepsy, pain, dyskinesia, tremor, dystonia, major depression and obsessive compulsive disorder (OCD). The exact mechanism of action is unknown and it is assumed that all neurons within a given volume are being activated. Because the cell type activated can be very different and sometimes non-neuronal (Nam et al., 2016), optical stimulation of the same volume might not perform an equivalent job. Epilepsy is presently the object of many attempts to apply neuro-modulation. This is justified by the large number of therapy resistant cases. However the pathophysiology of this condition remains obscure. The selectivity of optogenic stimulation might help sort out the pathophysiology and arrive at an efficient clinical treatment (Xu et al., 2016; Yekhlef et al., 2017). Also for conditions, such as retinal diseases, light stimulation would probably make the generation of meaningful visual perceptions easier in a retinal prosthesis (Nirenberg and Pandarinath, 2012; Al Atabany et al., 2013). Stimulation and sensing can be implemented exclusively with optical elements (Inagaki et al., 2017). A combination of techniques, such as electrophysiology and optogenetics (Chen et al., 2017) or micro-fluidics (Rubehn et al., 2013; McCall et al., 2017), can offer additional perspectives in feedback controlled systems.

## 8. Biocompatibility issues

### 8.1. Gene delivery

In a standard optogenetics protocol the channelrhodopsin gene is delivered by viral vector injection. However, as already mentioned, innate immunity and antigen-specific adaptive immune responses against vector-derived antigens could reduce the efficacy and stability of the gene transfer when this technique is translated to the clinic (for an overview, see Bessis et al., 2004; Nayak and Herzog, 2009). In the case of human gene therapy, AAV vectors are frequently put forward as optimal solutions due to their good safety profiles (Carter, 2005). Innate immune response are limited, but have been observed at high doses or with specific serotypes (Lowenstein et al., 2007; Hadaczek et al., 2009). Adaptive immune responses, on the other hand, are more common. Anti-AAV antibodies are predominantly directed at the vector's capsid protein (Mingozzi and High, 2013). Neutralizing antibodies constitute one of the main challenges of successful gene delivery, as they impact transfection efficiency in animal (Arruda et al., 2010; Haurigot et al., 2010; Jiang et al., 2013) and clinical studies (Manno et al., 2006), even at low titers (Scallan et al., 2006). Especially persons with pre-existing anti-AAV antibodies would be at a disadvantage, though there is a difference in neutralizing effect depending on the serotype (Xiao et al., 1999). Other studies in the brain (Lo et al., 1999; Mastakov et al., 2002), muscle (Kay et al., 2000) and retina (Anand et al., 2002) revealed, however, no relation between the presence of the anti-capsid antibodies and the amount of transgene expression.

At present, it is seems too early to choose between systemic vs. local viral vector application. Systemic application of viral vectors could be favorable in the case mechanisms of DBS turn out to be more delocalized. On the other hand, in this case the volume of illumination can turn out to be the performance limiting factor. Localized application by the device itself may pose conflicting engineering requirements (e.g., microfluidics), which could decrease reliability.

### 8.2. Expression of the transgene

Besides vector-specific antibodies, the body might also mount an immune response against the transgene itself. Transgene-specific immune responses are dependent on the target organ, the administration route and dosage (Toromanoff et al., 2010; Mingozzi and High, 2013). Target tissue such as the liver display a high level of tolerance to the transgene product (LoDuca et al., 2009), while muscle (Ross et al., 2006) is more prone to activation of the immune system. In the case of neural tissue, opsins do not seem toxic to human neurons in the brain or retina (Busskamp et al., 2010; Valtcheva et al., 2016). However, high levels of opsin expression might still induce cell death (Klein et al., 2006), alteration in electrical membrane properties or form aggregates in neurons (Gradinaru et al., 2008; Zimmermann and Dours-Zimmermann, 2008; Diester et al., 2011), while no observable damage has been reported (Han et al., 2009). The diversity in responses points to the difficulty of determining the correct levels of expression (Allen et al., 2015; Jarvis and Schultz, 2015). Therefore, to counter potential toxicity, different modification strategies have been developed, leading to safe expression even at high titers (Gradinaru et al., 2008). Finally, as in any gene therapy case, there is a risk that the opsin might insert randomly into the genome and thereby result in oncogenesis (Hacein-bey abina et al., 2008).

### 8.3. Bio-mechanical device compliance

The light-delivering device can either be implanted into or remain external to the brain. The necessity of opening a pathway in the first case bears the risk of tissue damage due to the insertion or chronic presence in the body as with any implantable device (for a review, see Prodanov and Delbeke, 2016a). Acute vascular damage can be controlled to some extent by the shape of the implant and the surgical trajectory; while the amount of micromotion is a function of the implant inertial properties and the mode of attachment to the skull. Both parameters are difficult to modify, once an application has been chosen. To prevent mechanical damage, the device should be as small as possible, while providing sufficient light to the target deep brain structures. For this purpose, multiple solutions have been proposed: for example, novel probe designs or adaptations of high-density fiber arrays (Zorzos et al., 2010; Han, 2012; Ozden et al., 2013; Hoffman et al., 2015).

### 8.4. Optical stimulation

Light absorbed by brain tissue can produce non-specific effects due to heating or photodamage. Heating may alter the activity of neurons or even behavior (Moser and Mathiesen, 1996; Long and Fee, 2008). The risk of overheating constraints the power that can be used by the device and hence limits the illumination volume. A possible solution is to place the device further from the neurons of interest or even outside the brain and use red light in combination with red-shifted opsins (Lin et al., 2013).

Whereas most of the optogenetic protocols do not induce excessive tissue damage (Gysbrechts et al., 2016), heating effects were also observed at standard light powers (Christie et al., 2013; Stujenske et al., 2015) and even in non-transduced cells (Han, 2012). On the other hand, heat itself has been used as well to perform optical stimulation of wild-type cells, using infrared pulses (Shapiro et al., 2012; Carvalho-de Souza et al., 2015). The light might also have non-thermal effects on neural tissue. Light-sensitive pathways naturally present in cells could produce unwanted effects to both targeted and surrounding cells (Koyanagi et al., 2013; Cheng et al., 2016).

### 8.5. Long-term effects

Finally, a favorable acute response does not necessarily predict the chronic stimulation effects on neuronal tissue. Changes at single-cell and network level need to be considered when translating optogenetics to the clinic. Long-term exposure to light pulses has been known to plastically modify the behavior of transfected neurons, changing their response to stimulation (Schultheis et al., 2011; Lignani et al., 2013), or induce long-term potentiation (Zhang and Oertner, 2007). Some of these chronic effects may prove to be beneficial in a clinical context, leading for example to a reduction of the stimulation periods. On a single-cell level, changes in neuron morphology have been observed, due to the prolonged expression (Miyashita et al., 2013) or stimulation (Goold and Nicoll, 2010; Grubb and Burrone, 2010).

Four main aspects contributing to compromised long-term compliance can be outlined:

- acute vasuclar damage including hemorrhage;
- micromotion of the implant;
- localized blood-brain barrier (BBB) breakdown;
- formation of reactive oxygen species (ROS, i.e., oxidative stress).

The disruption of BBB leads to the deposition of plasma proteins foreign to the CNS, such as albumin, globulins, fibrin/fibrinogen, thrombin, plasmin, complement (Kozai et al., 2015). The vascular damage is accompanied by fluid displacement, dragging of the blood vessels and eventual hemorrhage observed after implantation (Bjornsson et al., 2006). Hemorrhages have been shown to be particularly detrimental for long term recording (Stensaas and Stensaas, 1976; Turner et al., 1999; Grand et al., 2010). The cerebral vasculature is particularly susceptible to the action of ROS, which is of great importance since cerebral endothelial cells play a major role in the creation and maintenance of BBB (review in Obermeier et al., 2013). Such BBB dysfunction can result in an imbalance of ions, transmitters and metabolic products in the interstitial fluid, causing abnormal neuronal activity. In the implantation setting ROS can be linked to the catalytic function of *Fe*^3+^ present in the blood clot, which can lead to formation of stationary diffusion-limited concentration gradients around the implant (Prodanov and Delbeke, 2016b).

## 9. Neuromodulation

The clinical application of optogenetic systems (i.e., optical brain stimulators) will require much more attention for the target selection, stimulus characteristics (Shiri et al., 2017; Weible et al., 2017) and therapy regimen than has been the case hitherto. Within the framework of DBS, several different neural targets are still being proposed for electrical neurostimulation. Cortical stimulation is being considered for pain (Liu and Tao, 2016) and epilepsy as well as for totally different applications such as providing sense of touch to prosthetic hands (Nghiem et al., 2015).

### 9.1. Critera for clinical acceptance

Refractory epilepsy and Parkinson's disease present different cases for an eventual application of optogenetics. While the Parkinson's disease is an established target for DBS since 1997, long-term efficacy of electrical stimulation for refractory epilepsy is still an ongoing investigation. Despite maximal antiepileptic drug therapy, more than 30% of patients with epilepsy suffer from persistent seizures, while up to 40% of patients are not candidates for surgical resection (Halpern et al., 2008). The SANTE trial^3^ fond out that the median percent seizure reduction from baseline at 1 year was 41%, and 69% at 5 years. The long-term follow-up of anterior thalamic nucelus (ANT) DBS showed sustained efficacy and safety in a treatment-resistant population (Salanova et al., 2015). The readers are directed to the recent review of Laxpati et al. (2014) for a summary of the clinical trials in epilepsy.

While for Parkinson's disease an optogenetic stimulator must demonstrate superiority to eventually become an accepted therapy, in epilepsy the target can be somehow lowered to non-inferiority. This indicates that optogenetic modulation of refractory epilepsy could in fact be achieved first.

### 9.2. Alternative realization

Optogenetics also offers the possibility to work with cells other than neurons (Cho et al., 2016). Systems combining optic simulation and electrical recording have been developed (Wang et al., 2012; Rubehn et al., 2013; Hoffman et al., 2015, 2016; Naughton et al., 2016; Segev et al., 2017). Targets have not been limited to the brain and include the spinal cord and peripheral nerves (VNS for epilepsy and depression, motor nerves for various palsies, hypoglossal nerve for sleep apnoea and many more). For some peripheral nerves, optic stimulation could be realized without implant or transfection (Maimon et al., 2017). Alternatively, non invasive techniques are being developed with Transcranial Direct Current Stimulation (tDCS), transcutaneous VNS (tVNS), Repetitive Transcranial Magnetic Stimulation (rTMS) and others.

### 9.3. Limitations

Despite its acceptance, there is still much room for progress in the practice of DBS—for example, by providing bi-directional (i.e., including sensing) interfaces, which would open the path for closed-loop approaches and titration of stimulation. Neural tissue does not necessarily provide a stable response to chronic stimuli (McCreery et al., 1997). Poorly controllable brain plasticity can turn out to be an ally or an enemy in chronic applications. Along the same line, cellular biology homeostasis (Sinnen et al., 2017) should be considered. It is known that long term potentiation or depression (LTP, LTD) (Nabavi et al., 2014; Correia et al., 2017) can be induced by some stimulation conditions. Specific strategies might be essential in the control of epileptic activity (Ching and Ritt, 2013). Cell-type and even sub-cellular specificity hold the promise for real progress (Dvorzhak et al., in press) but we lack the knowledge to benefit from such developments. Also stimulus phase timing (Witt et al., 2013) can be important. Appropriate stimulation can shift the excitability of the cortex (Heitmann et al., 2017). Stimulation regimens are important (Shiri et al., 2017) but mostly established on empirical basis because of a lack of neurophysiological and pathophysiological understanding (Gradinaru et al., 2009; Dvorzhak et al., in press). Safe limits for the various stimulation parameters have not been firmly established. Despite the brain being essentially a control system, most rational explanations are still in terms of a simple balance between excitatory and inhibitory influences. Closed loop system are being presented (Grosenick et al., 2015) but they still represent “event-triggered” stimulation rather than network integrated devices.

### 9.4. Possible applications

DBS is a recognized therapy in Parkinson's disease and it has also benefited some cases of major depression. An optogenetics equivalent could do equally well or perhaps even better but there is no clinical evidence yet. Present findings point to a number of pathways along which optogenetics is likely to play a role in the near future, namely in the treatment of epilepsy (Mantoan Ritter et al., 2014). With the assumption that various issues related to genetic manipulations of adult patients can be resolved, optogenetics could soon become an alternative to DBS (Karas et al., 2013) in the treatment of *Parkinson's disease* (Beitz, 2014) and *epilepsy* (see for example Boon et al., 2007; Cox et al., 2014). Many more conditions might soon enter the therapeutic perspective including Alzheimer's disease (Kastanenka et al., 2017), the cochlear prosthesis (Richardson et al., 2017), visual pathologies (Scholl et al., 2016; Sengupta et al., 2016) and perhaps cardiac (Entcheva and Bub, 2016) or other non-neural conditions.

## 10. Conclusions and outlook

Presented brief overview of the perspectives of optogenetics for DBS and neuromodulation leads to the following conclusions:

On the first place, the term “optogenetics” cannot be reduced to a single method or technique but can be broken down into a number of components that could be used independently of assembled in various combinations leading to a very broad spectrum of exciting therapeutic perspectives. A bright future for such applications can be foreseen. Optogenetics thus appears as a challenge well worth the substantial research effort that it still requires.

Secondly, safety of viral vectors is not a roadblock toward human application of optogenetics. There is already sufficient experience with clinical trials and level of maturity of viral vector technology. Despite the major input of technology in the field, it also appears that a lack in fundamental knowledge remains a major obstacle to progress (Jarvis and Schultz, 2015). Investing massively in applied trials while neglecting basic science might not be the most cost-effective way on the long run. Safety, optimization and chronic applicability would surely benefit from such a basic knowledge-first based approach.

Thirdly, attempts to apply optogenetics immediately confront researchers with the large gaps in our knowledge of the brain. Clearly, trials based only on partial understanding will lead to inefficiency and increasing risks as already shown by the early failures in gene therapy. The shortest path toward successful optogenetics applications appears to be to focus on fundamental research on mechanisms of DBS.

Optogenetics itself seems to offer the necessary tools to perform the experimental brain studies that are so badly needed. Progress in the field precisely identifies the function of specific populations of neurons in a complex anatomical substrate. Chronic clinical evaluation is necessary before it can be stated that initial results will be maintained in time. Long term evolution of the therapeutic effects, however can turn out either way. It may be either favorable or leading to inefficiency and side effects.

Optogenetics can be considered as a case demonstrating the value of exploratory research. This is clearly recognized by Deisseroth (2015), who further admits that pioneering optogenetic experiments were not suitable to typical grant programs focusing on a disease state, on a translational question, or even on solidly justified basic science.

Finally, optogenetics provides a large variety of tools, which enable further fundamental research. On the other hand it also raises challenges because of its transdisciplinary character. The extraordinary therapeutic breakthroughs that can be foreseen, the sheer number of conditions for which an application seems ultimately possible and the accumulating evidence balances the hurdles described in the preceding sections.

## Author contributions

All authors listed have made a substantial, direct and intellectual contribution to the work, and approved it for publication.

### Conflict of interest statement

The authors declare that the research was conducted in the absence of any commercial or financial relationships that could be construed as a potential conflict of interest. The reviewer WW declared a past co-authorship with one of the authors JD to the handling Editor.

## Footnotes

^1^Conditioned on prior gene transfection by viral vectors or other means.

^2^International Neuromodulation Society: http://www.neuromodulation.com/about-neuromodulation.

^3^Stimulation of the Anterior Nucleus of the Thalamus for Epilepsy trial (SANTE; NCT00101933, Medtronic, Minneapolis, MN, USA).
